# Editorial: The Use of Bioactive Materials in Caries Management

**DOI:** 10.3389/froh.2022.832285

**Published:** 2022-03-08

**Authors:** Mary Anne Melo, May Lei Mei, Kai Chun Li, Hamdi Hosni Hamama

**Affiliations:** ^1^University of Maryland School of Dentistry, Baltimore, MD, United States; ^2^Department of Oral Rehabilitation, Faculty of Dentistry, University of Otago, Dunedin, New Zealand; ^3^Department of Operative Dentistry, Faculty of Dentistry, Mansoura University, Mansoura, Egypt

**Keywords:** dental caries, prevention, bioactive, dental materials, oral health

Contemporary caries management philosophy has changed from the traditional surgical approach to a medical model [1]. This approach often includes dietary analysis and advice, oral hygiene instruction, placement of fissure sealants, and the use of bioactive materials, including fluoride therapy, calcium phosphate products, peptides, bioactive glass and antimicrobial agents. Bioactive parts of the materials interact positively with the oral environment and promote caries management.

In recent years, there has been a great deal of emerging conservative approaches in caries management. This Research Topic is a dedicated platform to consolidate bioactive materials-related research and identify new potential strategies in caries management.

In the Research Topic “The Use of Bioactive Materials in Caries Management,” studies related to bioactive materials including fluoride, calcium phosphate products, peptides, antimicrobial agents, remineralizing agents on caries management were discussed.

Streptococcus mutans (S. mutans) has been identified as one of the leading role players in developing dental caries. S. mutans has been presented as one of the most virulent bacterial species in the caries process [3, 4] and has been implicated in the progression of dental caries. The metabolism of carbohydrates by S. mutans results in its adherence to the tooth structure and subsequent high aciduricity and acidogenecity. Plaque samples have been shown to contain high levels of L-lactic isomer that is the main acid produced by S. mutans. Glass ionomer cements has become the first choice restorative material for high caries risk patients because its inhibitory influence on S. mutans. Mulder et al. investigated the optical density of Streptococcus mutans (S. mutans) at 450 nm (OD450 nm) as well as the change in surface roughness of three commercially available chitosan- and nanodiamond-modified glass ionomers. Only the chitosan modifications showed an increase in the surface roughness after 24 h of exposure to the *S. mutans*.

Garcia et al. aimed to evaluate dental bioactive resins' wear behavior and surface quality under a simulated chewing model and compare them with a resin without bioactive agents. Bioactive materials can reduce caries lesions on the marginal sealed teeth by releasing ions, such as calcium, phosphate, fluoride, zinc, magnesium, and strontium [2]. The presence of such ions affects the dissolution balance of hydroxyapatite, nucleation, and epitaxial growth of its crystals. Previous studies mainly focused on the ion-releasing behavior of bioactive materials. Little is known about their wear behavior sealed tooth under mastication.

Unlike other fluoride-based caries preventive agents, silver diamine fluoride (SDF) can simultaneously prevent and arrest coronal and root dentine caries [3]. Looking to discuss the global policies, guidelines, and relevant information on utilizing SDF for caries management, Gao et al. provide an overview of SDF use in different countries and allow dental professionals to obtain a general idea about SDF use worldwide. The authors concluded that at least two ongoing regional-wide large-scale oral health programs using SDF as one of the components to manage dental caries in young children (one in Hong Kong and one in Mongolia). Because SDF treatment does not require caries removal, it is simple, non-invasive, and inexpensive. Thus, SDF is a valuable strategy for caries management in young children, older adults, and patients with special needs. In addition, to reduce the risk of bacteria or virus transmission in dental settings, using SDF as a non-aerosol-producing procedure should be emphasized under the COVID-19 outbreak.

We hope that this work set will stimulate further research on the development of bioactive materials for dental applications by providing an overview of the latest and most exciting advances in emerging approaches for bioactivity. In addition, this topic may help readers to understand the basics and latest developments in this field.

## Author Contributions

MMel, MMei, KL, and HH contributed to the writing of the manuscript. All authors contributed to the article and approved the submitted version.

## Conflict of Interest

The authors declare that the research was conducted in the absence of any commercial or financial relationships that could be construed as a potential conflict of interest.

## Publisher's Note

All claims expressed in this article are solely those of the authors and do not necessarily represent those of their affiliated organizations, or those of the publisher, the editors and the reviewers. Any product that may be evaluated in this article, or claim that may be made by its manufacturer, is not guaranteed or endorsed by the publisher.
